# *Salmonella* Impacts Tumor-Induced Macrophage Polarization, and Inhibits SNAI1-Mediated Metastasis in Melanoma

**DOI:** 10.3390/cancers13122894

**Published:** 2021-06-09

**Authors:** Christian R. Pangilinan, Li-Hsien Wu, Che-Hsin Lee

**Affiliations:** 1 Department of Biological Sciences, National Sun Yat-sen University, Kaohsiung 80424, Taiwan; d072010003@nsysu.edu.tw (C.R.P.); d082010004@nsysu.edu.tw (L.-H.W.); 2 Department of Medical Research, China Medical University Hospital, China Medical University, Taichung 40402, Taiwan

**Keywords:** *Salmonella*-mediated tumor therapy, melanoma, metastasis, macrophage polarization

## Abstract

**Simple Summary:**

Cancer treatment using *Salmonella* is deemed a viable option since *Salmonella* impedes expression of proteins relevant to tumor survival and progression. In essence, the present study investigated the effect of *Salmonella* on melanoma metastasis. We found that *Salmonella* reduces Akt/mTOR activity, resulting in downregulation of SNAI1—an EMT inducer vital for cell migration. Furthermore, *Salmonella* increases HMGB1 secretion in tumors, thereby influencing macrophage reprogramming toward an M1-like phenotype. We propose that these two processes, coaxed by *Salmonella*, work concurrently to prevent melanoma metastasis.

**Abstract:**

Targeting metastasis is a vital strategy to improve the clinical outcome of cancer patients, specifically in cases with high-grade malignancies. Here, we employed a *Salmonella*-based treatment to address metastasis. The potential of *Salmonella* as an anticancer agent has been extensively studied; however, the mechanism through which it affects metastasis remains unclear. This study found that the epithelial-to-mesenchymal transition (EMT) inducer SNAI1 was markedly reduced in *Salmonella*-treated melanoma cells, as revealed by immunoblotting. Furthermore, wound healing and transwell assays showed a reduced in vitro cell migration following *Salmonella* treatment. Transfection experiments confirmed that *Salmonella* acted against metastasis by suppressing protein kinase B (Akt)/mammalian target of rapamycin (mTOR) signaling, which in turn inhibited SNAI1 expression. Since it is known that metastasis is also influenced by inflammation, we partly characterized the immune infiltrates in melanoma as affected by *Salmonella* treatment. We found through tumor-macrophage co-culture that *Salmonella* treatment increased high mobility group box 1 (HMGB1) secretion in tumors to coax the polarization of macrophages in favor of an M1-like phenotype, as shown by increased inducible nitric oxide synthase (iNOS) expression and Interleukin 1 Beta (IL-1*β*) secretion. Data from our animal study corroborated the in vitro findings, wherein the *Salmonella*-treated group obtained the lowest lung metastases, longer survival, and increased iNOS-expressing immune infiltrates.

## 1. Introduction

Melanoma is a malignant tumor regarded as the most fatal amongst the known forms of skin cancer [1]. Metastatic spread of most cancers, including melanoma, generally leads to high mortality rate despite available interventions [1,2]. Cancer metastasis occurs when cancer cells gain the potential to migrate through the circulation, extravasation, and eventually invasion of distant tissues [2,3]. These phenomena are directed by the expression of various transcription factors, including members of the Snail family, particularly SNAI1, a known regulator of the epithelial-to-mesenchymal transition during early development [4]. In cancer, SNAI1 expression has been implicated in high-grade malignancies and correlated with poor prognosis, as it is one of the drivers of cancer progression to metastasis [5,6,7]. The elevated expression of SNAI1 in melanoma changes the expression of genes with regulatory functions in epithelial-to-mesenchymal transition (EMT) [8]. SNAI1 expression was reported to be mediated by several signaling mechanisms, including phosphoinositide 3-kinase (PI3K)/protein kinase B (Akt) signaling, which is known to modulate behavioral phenotypes in tumors such as anti-apoptosis, drug resistance, and metastasis [9,10].

Tumor-intrinsic SNAI1 not only has direct effects on cell migration and invasion but also impacts the tumor microenvironment by altering tumor secretomes, thereby increasing the tendency of tumors to facilitate metastatic processes [11]. An inflammatory tumor microenvironment provides the necessary elements for the progression of cancer by influencing tumor-associated macrophages (TAMs) to support tumor survival and metastasis [12]. Classically activated TAMs or M1-like macrophages undermine tumor development and progression, whereas alternatively activated (M2-like) TAMs positively influence metastasis by promoting immunosuppression, angiogenesis, and extracellular matrix (ECM) remodeling [13]. The literature suggests that the polarization of macrophages into an M1-like phenotype is commonly stimulated by lipopolysaccharide (LPS), interferon-gamma (IFN-*γ*), and tumor necrosis factor-alpha (TNF-*α*) [14,15], and a number of studies also suggest the involvement of high mobility group box 1 (HMGB1) in activating innate immunity and M1-like macrophage reprogramming [16,17]. On the other hand, the M2-like phenotype is stimulated by interleukins such as IL-4, IL-10, and IL-13 [14,15]. Macrophage polarization into either the M1 or M2 phenotype is a consequence of two antagonistic pathways utilizing arginine: (a) the inducible nitric oxide synthase (iNOS) pathway, an indicator of the M1-like macrophage that generates citrulline and NO from arginine metabolism, and (b) the arginase (ARG) pathway in the M2-like macrophage that produces ornithine and urea [18].

Although there exist several drug candidates and treatment options to address cancer metastasis, the overall success, not to mention the affordability, remains a challenge. There is now an increasing interest in the use of *Salmonella*—a facultatively anaerobic, Gram-negative bacteria—in cancer therapy over conventional treatments for several reasons: *Salmonella* is quite motile, and it can preferentially target and penetrate tumors and disperse evenly within the hypoxic core with a tumor-to-normal tissue ratio of about 1000–10,000 to 1 [19,20]. Apart from its targeting potentials, *Salmonella* has been known to alter the overall gene expression landscape of the cell it invades, and thus in turn influences the activation and/or suppression of various signaling pathways to control cell behavior [19,20,21]. Despite the increasing number of reports on *Salmonella*-mediated cancer therapy, further investigations are essential to explore its innate ability that is vital in addressing various cancer types, including melanoma.

The present study looked into the ability of *Salmonella* to alter the gene expression profile of tumor cells. Here, we used *Salmonella enterica* serovar Choleraesuis—an attenuated vaccine strain—as an anti-melanoma metastasis agent. In particular, we aimed to investigate how *Salmonella* influences the behavioral phenotype of melanoma cells and its microenvironment in relation to cell migration and metastasis.

## 2. Results

### 2.1. Salmonella Suppresses Akt/mTOR Activity and SNAI1 Expression in Melanoma

**Figure 1:**
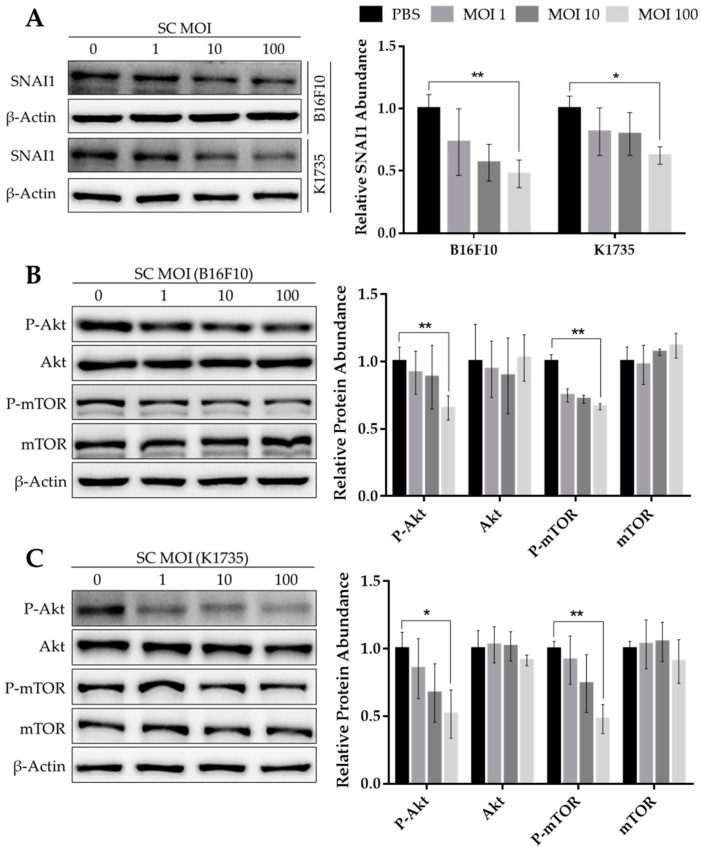
Expression levels of SNAI1 and Akt/mTOR phosphorylation following *Salmonella* treatment. Protein levels of (**A**) SNAI1, (**B**,**C**) Akt, P-Akt, mTOR, and P-mTOR from Western blot (WB), normalized to *β*-Actin, relative to 0 M.O.I.; quantified band intensities are presented alongside each WB image. Mouse B16F10 and K1735 melanoma cells were seeded in 6-well plates with a seeding density of 3 × 10^5^ and 4 × 10^5^, respectively. Cells were treated with varying doses of *Salmonella* from 0–100 multiplicity of infection (M.O.I.) for about 90 min; cells were washed and cultured in serum-free DMEM with 50 µg/mL gentamicin. After 24 h, cells were lysed, and the lysates were subjected to Western blotting. Data are presented as mean ± SD from three independent experiments. Statistical significance was calculated using Student’s *t*-test. * *p* < 0.05, ** *p* < 0.01.

Substantial pieces of evidence demonstrate the role of the Snail family of transcription factors in regulating the epithelial-to-mesenchymal transition, or EMT—an essential process that facilitates cancer progression to metastasis [4]. Given that *Salmonella* influences gene expression in tumors by modulating Akt/mTOR and MAPK signaling [19], we examined the effect of *Salmonella* treatment on the expression of SNAI1 in a panel of melanoma cell lines, as SNAI1 was known to be regulated, in part, by Akt signaling [9,10]. Here, we found that increasing multiplicity of infection (M.O.I.) amplified the effect of *Salmonella* treatment on the reduction of SNAI1 protein in mouse B16F10 and K1735 cell lines, with maximal efficiency at 100 M.O.I. (Figure 1A). The same effect was observed in human melanoma cells after *Salmonella* treatment, showing a significant decrease in SNAI1 expression (Figure S1A,B). We then considered the signaling mechanism that may be involved in the downregulation of SNAI1 following *Salmonella* treatment. Previous reports showed that SNAI1 is regulated by the PI3K/Akt signaling pathway [9,10]. Interestingly, the same reduction pattern of SNAI1 brought about by *Salmonella* treatment was observed in the levels of Akt and mTOR phosphorylation, with an apparent drop at 100 M.O.I. in all cells tested (Figure 1B,C and Figure S1A,B); hence, these results indicate the probable action of *Salmonella* on SNAI1 by disrupting intracellular Akt/mTOR signaling.

### 2.2. Salmonella Inhibits Migrative Behavior of Melanoma Cells In Vitro

**Figure 2:**
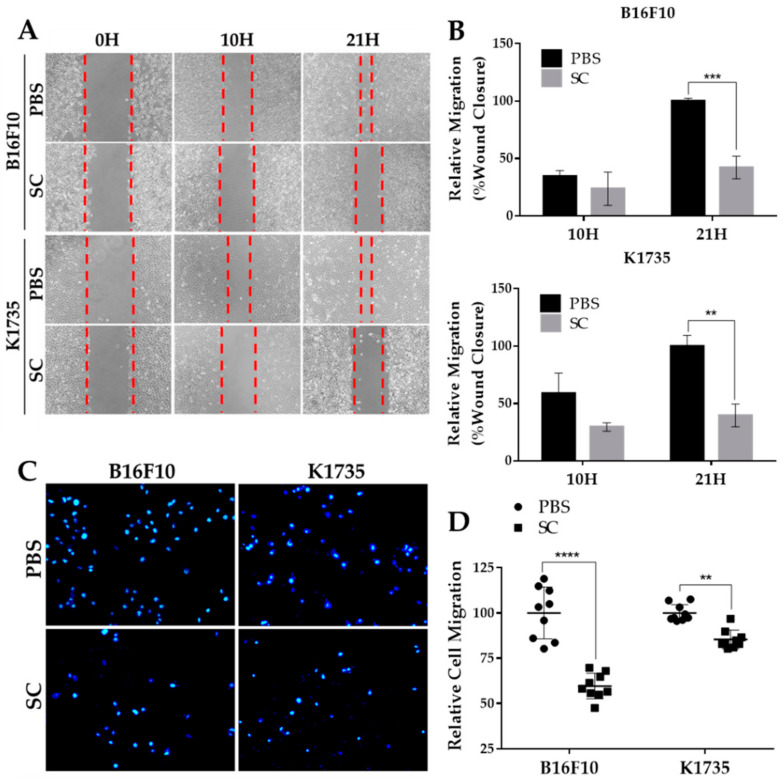
In vitro migration of *Salmonella*-treated melanoma cells. (**A**,**B**) Percent wound closure as relative migration in two melanoma cell lines after *Salmonella* treatment. Seventy (70) µL cell suspension was seeded (B16F10, 2 × 10^5^/mL; K1735, 3 × 10^5^/mL) into 2-well culture inserts positioned inside 12-well plates; the cells were treated with *Salmonella* 100 M.O.I. and photographed (100×) after 10 h and 21 h thereafter. (**C**,**D**) Relative number of migrating cells passing through the membrane of hanging culture inserts. Cell were pretreated with either PBS or *Salmonella* 100 M.O.I. and seeded (200 µL of B16F10, 1 × 10^5^/mL or K1735, 1.5 × 10^5^) into transwell hanging culture inserts placed inside a 24-well plate; cells were fixed with 3% paraformaldehyde, stained with DAPI, and counted under 200× magnification. Data are presented as mean ± SD. Each experiment was repeated thrice with similar outcomes. Statistical significance was calculated using Student’s *t*-test; ** *p* < 0.01, *** *p* < 0.001, **** *p* < 0.0001.

The expression of EMT factor SNAI1 in melanoma and other cancers correlates with tumor aggressiveness, as it positively regulates various protumor genes, including metastasis inducers [8,9,10]. Since *Salmonella* treatment significantly reduced SNAI1 expression (Figure 1A), we assessed its biological impact on melanoma cell migration. We first employed a wound healing assay to observe the percentage of wound closure as migration distance in B16F10 and K1735 cell lines. As seen in Figure 2A,B, cell migration distance was significantly decreased in both melanoma cell lines following *Salmonella* treatment at 100 M.O.I. compared with the PBS control. To rule out the possible interference of cell proliferation in wound healing, we used a transwell assay as our second approach to assess and validate cell migration. We found that after *Salmonella* treatment, the number of migrating cells across the membrane of the hanging cell culture inserts was significantly lower than the control (Figure 2C,D). These results indicate that *Salmonella* can prevent cell migration, an essential phenomenon that prompts tumor metastasis.

### 2.3. Salmonella Treatment Reverses the Constitutively Active Akt-Induced SNAI1 Overexpression and Tumor Cell Migration

**Figure 3:**
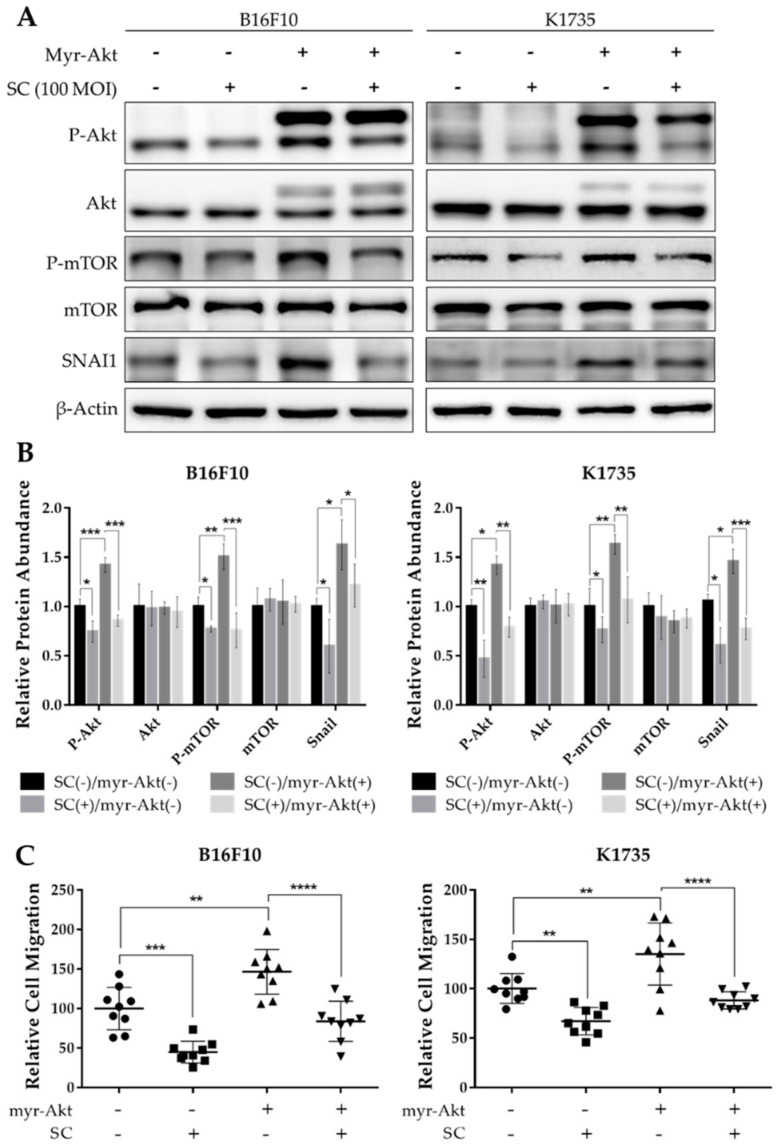
*Salmonella* inhibited SNAI1-mediated migration through Akt/mTOR signaling. Melanoma cells were seeded in 6-well plates at 70–80% confluency and transfected with constitutively active Akt plasmid for 16 h, re-seeded, and treated with PBS or *Salmonella* 100 M.O.I. thereafter. (**A**) Western blot images quantified in (**B**) showing Akt, P-Akt, mTOR, P-mTOR, and SNAI1; values are normalized to *β*-Actin, relative to control (SC(−)/myr-Akt(−)). (**C**) Relative number of migrating cells in the transwell assay; transfected and non-transfected melanoma cells were pre-treated with PBS or *Salmonella* 100 M.O.I. prior to seeding. Cells were fixed with paraformaldehyde, stained with DAPI, and counted under 200× magnification. Data are presented as mean ± SD. Each experiment was repeated three times with similar outcomes. Statistical significance was calculated using ANOVA and Tukey’s multiple comparison test; * *p* < 0.05, ** *p* < 0.01, *** *p* < 0.001, **** *p* < 0.0001.

Akt signaling has since been implicated in regulating various cellular processes in tumors, such as survival and proliferation, and more importantly, tumor invasiveness and metastatic dissemination [22]. Given our findings that *Salmonella* treatment suppresses Akt/mTOR activity, we induced constitutive and enhanced activation of Akt by transfecting B16F10 and K1735 cells with myr-Akt plasmids to assess whether Akt signaling is indeed involved in *Salmonella*-induced regulation of SNAI1 expression. We found that when transfected with Akt plasmid, not only was the phosphorylation of both Akt and mTOR enhanced, but the SNAI1 expression was also increased significantly. Interestingly, though, *Salmonella* treatment overturned the enhanced Akt/mTOR activity and increased SNAI1 expression in Akt-transfected melanoma cells (Figure 3A,B). Furthermore, we examined the cell migration of Akt-transfected B16F10 and K1735 cells and found that there was a significant increase in the number of migrating cells compared with the PBS control and that, as shown in Figure 3C, the migration rate was again decreased following *Salmonella* treatment. At this point, we can surmise that *Salmonella* inhibits SNAI1 expression through the Akt/mTOR axis to prevent cell migration in vitro.

### 2.4. Salmonella-Treated Melanoma Cells Reprogram Macrophage into M1-Like Phenotype

**Figure 4:**
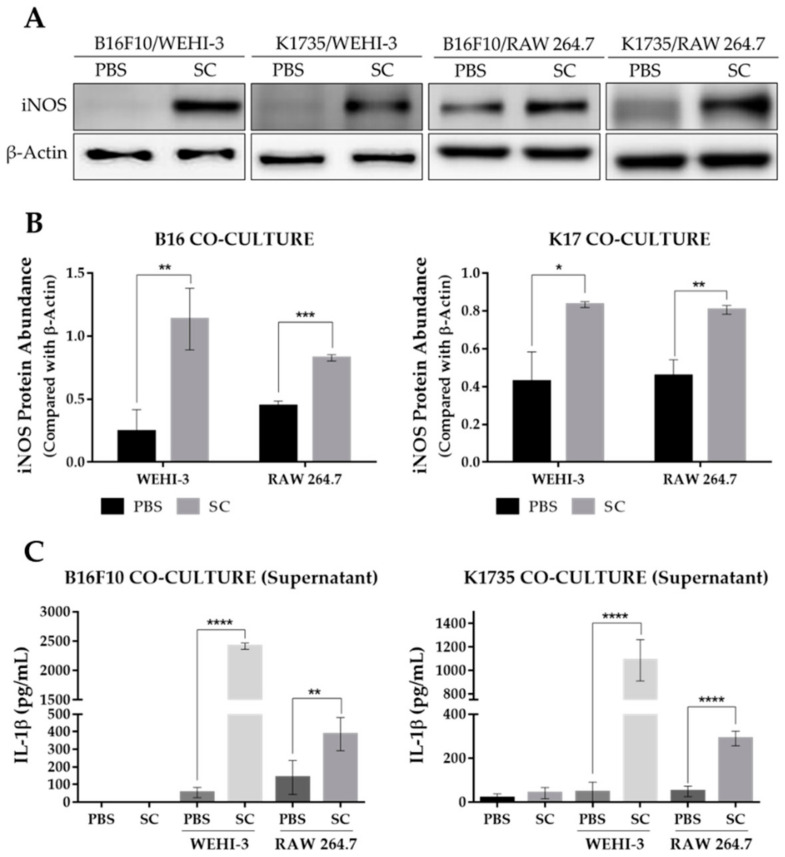
Expression of M1-like macrophage polarization markers as affected by *Salmonella* treatment. (**A**) Western blot images quantified in (**B**) showing protein levels of iNOS in WEHI-3 and RAW 264.7 cells, normalized to *β*-Actin. (**C**) Secreted IL-1*β* levels in culture supernatants from tumor-macrophage co-culture. WEHI-3 and RAW 264.7 cells were co-cultured with PBS or *Salmonella* pre-treated melanoma cells using 0.4 µm transwell hanging culture inserts and incubated for 16 to 24 h; macrophage cells and the conditioned media were then separated for immunoblotting and ELISA. Data are presented as mean ± SD. Each experiment was repeated three times with similar result. Statistical significance was calculated using Student’s *t*-test; * *p* < 0.05, ** *p* < 0.01, *** *p* < 0.001, **** *p* < 0.0001.

**Figure 5:**
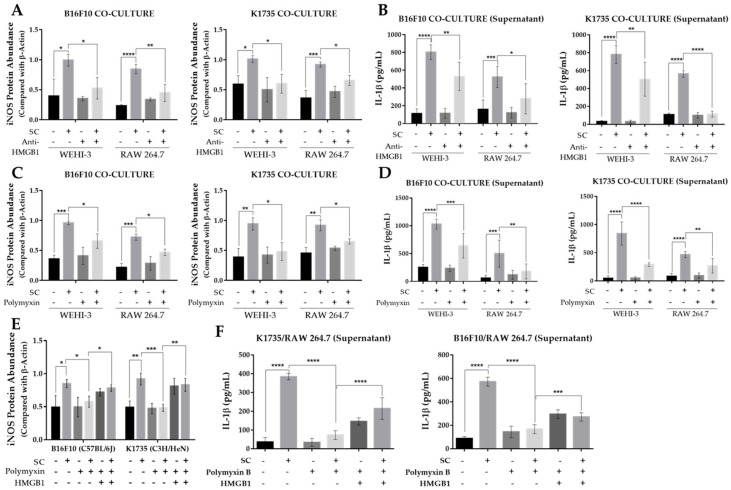
*Salmonella*-induced secretion of HMGB1 in melanoma is necessary for macrophage polarization. (**A**) Quantified band intensity from Western blot showing protein levels of iNOS in WEHI-3 and RAW 264.7 cells as affected by Anti-HMGB1 neutralizing antibody (2.5 µg/mL); protein signals were normalized to *β*-Actin. (**B**) Secreted IL-1*β* in HMGB1-neutralized culture supernatants from tumor-macrophage co-culture. (**C**) Quantified band intensity from Western blot showing protein levels of iNOS in WEHI-3 and RAW 264.7 cells as affected by Polymyxin B (10 µg/mL) treatment to neutralize LPS; protein signals were normalized to *β*-Actin. (**D**) Secreted IL-1*β* in LPS-neutralized culture supernatants from tumor-macrophage co-culture. (**E**) Quantified band intensity from Western blot showing rescued iNOS expression in WEHI-3 and RAW 264.7 by recombinant HMGB1 (5 ng/mL) treatment in LPS-neutralized tumor-macrophage co-culture. (**F**) Secreted IL-1*β* in LPS-neutralized culture supernatants from tumor-macrophage co-culture following recombinant HMGB1 treatment. WEHI-3 and RAW 264.7 cells were co-cultured with PBS or *Salmonella* pre-treated melanoma cells using 0.4 µm transwell hanging culture inserts and incubated for 16 to 24 h; cells were lysed, and the lysate was subjected to immunoblotting. Data are presented as mean ± SD. Each experiment was repeated three times with similar results. Statistical significance was calculated using ANOVA and Tukey’s multiple comparison test; * *p* < 0.05, ** *p* < 0.01, *** *p* < 0.001, **** *p* < 0.0001.

Tumor growth and progression are largely affected by an inflammatory tumor microenvironment. Inflammation within a tumor constitutes an immunosuppressive microenvironment, which is a trait primarily established by infiltrating immune cells, such as tumor-associated macrophages, or TAMs, that influence tumor growth and progression to metastasis [23,24]. As *Salmonella* affects the expression of various genes in tumor cells, including immune modulators [19,20,21], we determined the effect of an altered tumor secretome on macrophage polarization by using a tumor-macrophage co-culture system. Here, we used 0.4 µm hanging cell culture inserts for the co-culture system to prevent possible effects brought about by tumor-macrophage direct contact. We used WEHI-3 and RAW 264.7 cells to be co-cultured with PBS- or *Salmonella*-treated B16F10 or K1735 cells. The results show that pre-treatment of melanoma cells with *Salmonella* prior to macrophage co-culture generated an M1-like phenotype, as indicated by a significant increase in iNOS expression in WEHI-3 and RAW 264.7 cells (Figure 4A,B). M1 macrophages are known to produce certain cytokines not commonly produced by M2 macrophages such as IL-1*β*. Our co-culture experiment revealed that IL-1*β* secretion was significantly higher in groups involving *Salmonella* pre-treatment as measured using ELISA, whereas IL-1*β* was scarcely detected in groups without *Salmonella* pre-treatment (Figure 4C). Next, we investigated what coaxed the polarization of macrophages in the co-culture experiment. Known stimulants associated with polarization include LPS, IFN-*γ*, TNF-*α*, and HMGB1 [14,15,16,17]. Here, we measured HMGB1 secretion by Western blotting and ELISA analysis of culture supernatants from PBS- or *Salmonella*-treated melanoma cells. We found that extracellular HMGB1 was only detectable by Western blotting of culture supernatants at the highest *Salmonella* M.O.I. treatment. Although ELISA analysis showed that varying M.O.I. may trigger the release of HMGB1, only the highest M.O.I. treatment generated a significant quantity (Figure S2A). To confirm the involvement of tumor-secreted HMGB1 in macrophage polarization, we carried out the same co-culture experiment, this time supplemented with HMGB1 neutralizing antibody. The results show that anti-HMGB1 reversed the effects of *Salmonella* treatment on macrophage reprogramming (Figure 5A,B and Figure S3A). Specifically, the addition of anti-HMGB1 significantly reduced the expression of iNOS and IL-1*β* secretion in WEHI-3 and RAW 264.7 cells co-cultured with *Salmonella* pre-treated melanoma cells. To rule out the direct effect of LPS in macrophage reprogramming, we neutralized LPS in *Salmonella*-treated melanoma cells with 10 µg/mL Polymyxin B for at least 1 hr prior to co-culture. LPS neutralization resulted in significantly reduced iNOS expression and IL-1*β* secretion (Figure 5C,D and Figure S3B). Based on the findings so far, both extracellular HMGB1 and LPS seemed to affect M1-like phenotype activation. This led us to further investigate the relationship between HMGB1 and LPS in promoting polarization. This time, we neutralized LPS in *Salmonella*-treated melanoma cells using Polymyxin B and then measured HMGB1 in the culture supernatant. Surprisingly, extracellular HMGB1 was found to be significantly reduced after 24 h incubation in LPS-neutralized culture (Figure S2B). To verify whether HMGB1 is directly involved in macrophage reprogramming, we added recombinant HMGB1 in LPS-neutralized co-cultures. As expected, iNOS expression and IL-1*β* secretion was rescued in macrophages from the co-culture system following recombinant HMGB1 treatment despite the presence of Polymyxin B (Figure 5E,F and Figure S3C). Thus, these findings demonstrate the participation of HMGB1 from *Salmonella*-treated tumor cells in macrophage reprogramming.

### 2.5. Salmonella Increases Tumor-Infiltrating M1-Like Macrophages and Inhibits Lung Metastasis In Vivo

**Figure 6:**
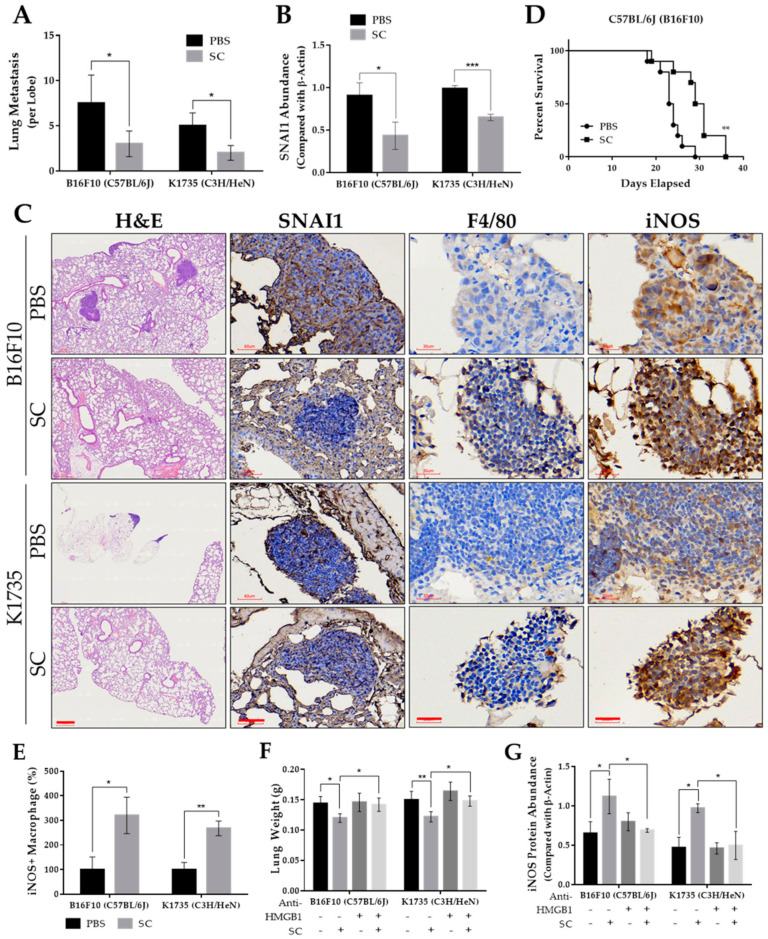
*Salmonella*-inhibited pulmonary metastasis in vivo. (**A**) Microscopically visible lung metastases per lobe of H&E-stained sections. (**B**) Quantified protein levels (Western blot) from metastatic tumor lysates, normalized to *β*-Actin. Lungs were harvested and the visible tumors were carefully dissected after 15 and 30 days post tumor injection with B16F10 and K1735 cells, respectively; *n* = 5. (**C**) H&E-stained lung tissue sections showing metastatic tumor nodules, and IHC-stained lung tissue using anti-SNAI1, anti-F4/80, and anti-iNOS; H&E scale bar, 300 µm (100×); SNAI1 IHC scale bar, 60 µm (200×); F4/80 and iNOS scale bar, 30 µm (400×). (**D**) Mouse survival after tumor inoculation (*n* = 10). (**E**) Number of iNOS+ infiltrating cells observed in IHC sections. (**F**) Lung weight after 15 and 30 days post tumor injection with B16F10 and K1735 cells, respectively; *n* = 5. (**G**) Quantified protein levels (Western blot) from metastatic tumor lysates, normalized to *β*-Actin. Data are presented as mean ± SD. Statistical significance was calculated using Student’s *t*-test (**A–E**) and ANOVA and Tukey’s multiple comparison test (**F**,**G**); * *p* < 0.05, ** *p* < 0.01, *** *p* < 0.001.

To substantiate our in vitro findings, we used two murine strains as melanoma models for the assessment of pulmonary metastasis. Figure S4A shows that the average lung weight of the *Salmonella*-treated group in each murine model was significantly lower than the PBS control. This can be explained by the presence of multiple tumor masses in the lungs as well as microscopically observed tumor nodules in the H&E-stained lung tissue sections of the PBS group (Figure 6A,C and Figure S4B). The observed reduction in metastatic activity can be accounted for, in part, by the downregulation of SNAI1 in the *Salmonella*-treated mouse group, as observed by the Western blotting of metastatic tumor lysates (Figure 6B and Figure S4C) and in IHC-stained lung tissue sections (Figure 6C). IHC staining further revealed a varying expression of macrophage polarization markers leaning toward M1-like infiltrates. In particular, the number of iNOS+ infiltrates were found to be higher in the *Salmonella*-treated group (Figure 6C,E). Further, we monitored mouse survival and found that in the B16F10 model, the *Salmonella*-treated group survived longer than the PBS control (Figure 6D). However, in the K1735 model, we terminated the experiment, as there were still no recorded deaths after 90 days post tumor inoculation. Nonetheless, the presence of metastatic lesions in the lungs was observed in the PBS control, as already mentioned. Lastly, we evaluated in a separate animal study whether the HMGB1 effect on M1-like macrophage infiltration and activation in relation to metastasis could also be seen in vivo. Neutralizing HMGB1 antibody simultaneously injected in mice along with PBS or *Salmonella* pre-treated tumor cells indeed impacted lung weight, an indication of lung metastasis (Figure 6F), consistent with the downregulation of iNOS (Figure 6G and Figure S4D). Altogether, these findings confirmed that the action of *Salmonella* against melanoma metastasis is rather intricate, involving multiple mechanisms such as targeting tumor SNAI1 expression, prompting macrophage reprogramming, and possibly other features that are open for further investigation.

## 3. Discussion

Metastasis has always been a major challenge for scientists and clinicians alike in combating aggressive cancers, including melanoma. It is an outcome of a complex interplay among the molecular pathways inherent to tumor cells, and the intricate interaction between tumor cells and their microenvironment [25]. These processes enable tumor tissue to shift from a state of dormancy to tumor progression by prompting angiogenic sprouting, migrative and invasive behavior, microenvironment modulation, and eventually the establishment of a distant secondary mass [2,3,4]. Effectively targeting metastasis will undoubtedly help improve the clinical outcome and survival of cancer patients. Despite the advances in drug development, most candidate drugs in pre-clinical and clinical trials explicitly highlight the effect in halting metastasis rather than the destruction of existing metastases [26]. Here, we employed *Salmonella*-based treatment as anti-metastatic agent. Our previous reports pointed out that *Salmonella* treatment significantly reduces tumor burden and prolonged survival of murine tumor models; the findings were attributed to the observed alteration of relevant signaling cascades such as AKT/mTOR/p70s6K pathway and p38-MAPK signaling axis, and varied expression of corresponding proteins such as P-glycoprotein, PD-L1, and Cx43, among others [19,27,28]. This led us to investigate the effect of *Salmonella* treatment on metastasis and its mediators.

**Figure 7:**
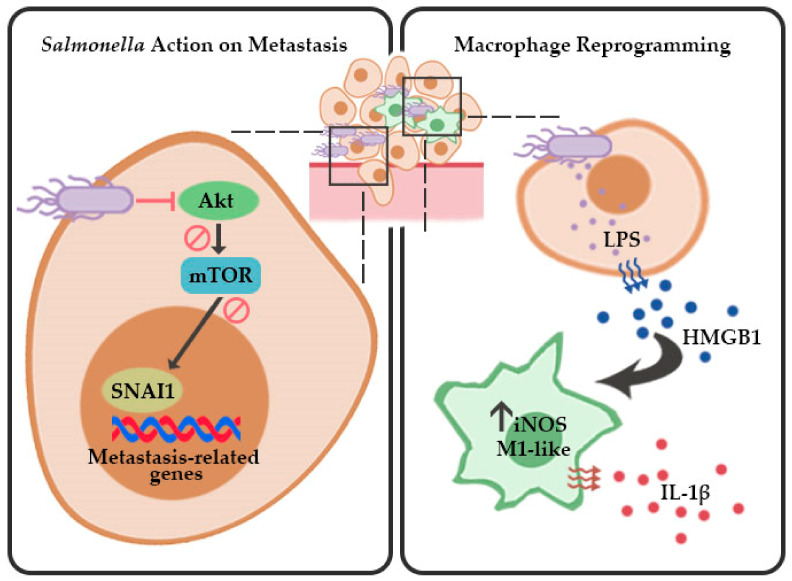
Proposed schematic model for *Salmonella*-mediated inhibition of metastasis and macrophage reprogramming in melanoma. *Salmonella* inhibits the EMT and migration factor SNAI1 by suppressing Akt/mTOR signaling and stimulates activation of M1-like macrophages by inducing HMGB1 secretion in melanoma.

It is known that SNAI1 acts as a key player in melanoma metastasis, as it affects a variety of tumor cell functions, and consequently, its expression correlates with poor clinical outcomes [29,30,31]. One vital feature of metastasis is the activation of EMT, which contributes to the enhanced migrative and invasive behavior of tumor cells [4,8]. We primarily attributed the reduction in migrative behavior in melanoma by downregulation of the EMT factor SNAI1, rather than as a direct outcome of the oncolytic effect of *Salmonella*. In our previous findings, the number of viable and proliferating cells was not significantly affected by *Salmonella* treatment at the same duration of infection [28]. Differential expression of EMT-related genes relevant to cell migration and ECM remodeling was found in both primary and metastatic clinical samples of melanoma [32]. Furthermore, snail proteins were found to be highly expressed in human tissue samples of cutaneous malignant melanoma [29]. Studies show that SNAI1 regulates EMT and metastasis-related proteins [8,33,34,35]. Among these proteins, the presence of SNAI1 elicits an increase in cyclin D1 and N-cadherin and causes downregulation of E-cadherin to induce EMT [33,34,35]. In addition, matrix metalloproteinases such as MMP-2 and MMP-9—MMPs are gelatinases primarily involved in ECM degradation and angiogenesis—were evidently controlled by SNAI1 [33]. These findings are in sync with the present study, as they provide a supplemental explanation corresponding to the phenomenon observed in our tumor models—the downregulation of SNAI1 by *Salmonella* led to the inhibition of migrative and metastatic behavior in melanoma, as illustrated in Figure 7. In a recent study, pharmacological targeting of Snail activity by blocking CBP/p300–Snail interaction demonstrated therapeutic benefits that prevented metastasis in a murine metastasis model [36]. Indeed, targeting SNAI1 is a step closer to eradicating cancer. However, the lack of an appropriate delivery system may prove challenging in addressing this disease. Interestingly, *Salmonella* harbors the innate potential to target SNAI1 based on our findings, not to mention that it is being explored through various means as a viable delivery vector to carry therapeutic genes [37], which may include oncolytic and antimetastatic factors. The use of *Salmonella* in cancer therapy has gained attention in recent years primarily because of its unique targeting ability, as previously mentioned.

We also investigated the upstream signals that affected SNAI1 expression in the present work. Initial findings showed that the phosphorylation of Akt and mTOR was directly proportional to the SNAI1 protein abundance following *Salmonella* treatment. This makes sense, since the experimental group demonstrating a more migrative phenotype in vitro also had high Akt/mTOR phosphorylation along with high SNAI1 expression. PI3K/AKT signaling—one of the most prominent pathways in melanoma pathogenesis—is identified as one of the culprits in the deregulation of many normal cellular functions such as apoptosis, proliferation, and cell migration [38]. These are accomplished through interfering signaling cascades, resulting in mTOR activation pertinent to tumor survival and progression [38,39,40]. The link connecting Akt/mTOR signaling with our *Salmonella*-mediated SNAI1 downregulation was confirmed by transfection experiments, wherein the constitutively active Akt-bearing cells showed a more migrative activity and higher SNAI1 expression, which were then reversed after *Salmonella* treatment (Figure 7). Likewise, a number of studies demonstrated that PI3K/Akt signaling is indeed one of the regulators of SNAI1, particularly in other types of cancer [9,10]. In addition, in a study by Fang et al. [35], PI3K/Akt signaling was necessary to prevent GSK-3*β*-facilitated Snail degradation in murine melanoma.

Inflammation is another aggravating factor within the tumor microenvironment that accelerates tumor progression to metastasis [12,13]. Considering that *Salmonella* altered the signaling cascades in melanoma, we theorized that the resulting reduction in metastasis was not solely due to the direct effect of SNAI1 downregulation on tumor-intrinsic mediators of metastasis but may also have been a consequence of the changes in the tumor inflammatory microenvironment. Previously, other groups showed that *Salmonella* increased infiltrates, shifting to the M1-like phenotype and its impact on tumor regression [41]. However, the phenomenon was described as a direct effect of *Salmonella* on immune infiltrates rather than as an outcome of *Salmonella*-influenced tumor–macrophage interaction. Here, we partly characterized the immune infiltrates in *Salmonella*-treated murine melanoma with emphasis on macrophage polarization into the M1-like phenotype. Our data indicate that *Salmonella*-treated melanoma influences reprogramming of macrophages into the M1-like phenotype, characterized by a significant increase in iNOS expression and IL-1*β* secretion. A number of studies demonstrate the role of the late inflammatory cytokine HMGB1 in macrophage reprogramming via TLR4 signaling [17,42]. Interestingly, we found that *Salmonella* treatment on melanoma cells triggered an increased secretion of HMGB1. In other cancer types, the depletion of SNAI1 causes changes in tumor secretome, specifically by increasing GM-CSF production, leading to an altered TAM polarization that is inclined toward M1-like macrophages [11]. We also looked into the possible role of extracellular TGF-*β*1, as an existing report suggested that TGF-*β* influences the activation of macrophages [43], and therefore might provide additional insight into how *Salmonella* impacts macrophage polarization in our experiments. However, we found that TGF-*β*1 was barely detectable in *Salmonella*-treated melanoma cells and even in the PBS control after 24 h incubation (incubation period used in most of our setups), which ruled out the participation of TGF-*β*1 in influencing macrophage polarization in our setup (Figure S5). Although we observed that the same experimental group showing SNAI1 downregulation also exhibited a high M1-like phenotype, we were unable to establish a link between Akt/mTOR/SNAI1 and HMGB1 secretion since we found no significant difference between the extracellular HMGB1 of Akt transfected melanoma cells and that of the control (Figure S2C). Nevertheless, our data suggest that polarization toward the M1-like phenotype is caused, in part, by extracellular HMGB1 from *Salmonella*-treated melanoma cells (Figure 7).

In a report from another group, flagellin derived from *Salmonella typhimurium* FliCi combined with MHC class II-restricted P10 peptide inhibited lung metastasis and activation of tumor-specific CD4(+) T cells in a murine melanoma model [44]. In another study, the *Salmonella* Typhi vaccine strain CVD 915, orally administered to BALB/c mice, elicited a T-cell mediated immunity by increasing IFN-*γ* and TNF production in the tumor, resulting in a reduced number of liver metastases [45]. Furthermore, an engineered *Salmonella typhimurium* secreting heterologous flagellin B (FlaB) was found to induce phenotypic and functional activation of M1-like macrophages via TLR4 instead of TLR5, which is the commonly known receptor for the FlaB ligand, which in turn suppressed tumor growth and inhibited metastasis [46]. In relation to this, our previous findings suggested the involvement of TLR4 in the *Salmonella*-mediated increase in immune infiltrates, including macrophages, neutrophils, and CD4+ and CD8+ T cells [47]. However, the macrophage infiltrates were not characterized, and the mechanism for the activation of each immune infiltrate was not given emphasis, and hence, was one of the purposes of the present study. The same strain used in the present study demonstrated changes in immune infiltrates, suggesting the action of *Salmonella* on CD4(+) and CD8(+) T cells by influencing PD-L1 expression, but not on TAMs [28]. This means that TLRs may be directly involved in macrophage reprogramming rather than CD4(+) and CD8(+) T-cell activation. This, however, needs further study. Considering that *Salmonella*-mediated therapy holds promise in addressing cancer, the progress in this line of research merits further investigation and translation into clinical trials. With this, there is no doubt that this innovative technology will find its way as one of the treatment options available for cancer in the near future, either as monotherapy or in tandem with other innovative strategies for a more efficient way to eliminate cancer.

## 4. Materials and Methods

### 4.1. Cell Lines, Bacteria, Plasmid, and Animals

The mouse B16F10, mouse K1735, mouse RAW 264.7, and human A2058 melanoma cell lines were maintained in Dulbecco’s modified eagle medium (DMEM) supplemented with 10% fetal bovine serum (FBS) and 1% penicillin/streptomycin. The WEHI-3 cell line was maintained in Iscove’s modified Dulbecco’s medium (IMDM) supplemented with 10% FBS, 0.05 mM 2-mercaptoethanol, and 1% penicillin/streptomycin. *Salmonella enterica* serovar Choleraesuis (ATCC 15480, Bioresources Collection and Research Center, Hsinchu, Taiwan), referred to as SC in this paper, was maintained on LB plates and propagated in LB broth for ≤16 h prior to use in the succeeding experiments. Constitutively active Akt plasmid, herein referred to as myr-Akt, was previously described [27,28]. C57BL/6J and C3H/HeN mice were procured from the National Laboratory Animal Center of Taiwan.

### 4.2. Western Blot Analysis

Cells were lysed in lysis buffer containing either protease inhibitors or phospho-protease inhibitors. Protein concentration was determined using Pierce™ bicinchoninic acid (BCA) protein assay (Thermo Fisher Scientific, Rockford, IL, USA). Proteins from cell lysates were electrophoresed through SDS-PAGE, transferred to nitrocellulose membranes (Pall Life Science, Glen Cove, NY, USA), and blocked in 5% milk for 1 h at room temperature. Blocked membranes were incubated with primary antibodies (Table S1) at 4 °C overnight, and with secondary antibodies for 2 h at room temperature; rabbit anti-mouse IgG-peroxidase antibody (Sigma Aldrich, St. Louis, MO, USA) and goat anti-rabbit IgG-peroxidase antibody (Sigma Aldrich, St. Louis, MO, USA) were used as secondary antibodies. A chemiluminescence system (T-Pro Biotechnology, New Taipei City, Taiwan) was used to visualize the signals and the exposures were acquired using a Fusion Imaging System (Vilber Lourmat, France). Signals were quantified using ImageJ software (NIH).

### 4.3. In Vitro Inhibition of Melanoma Cell Migration

The wound-healing assay using 2-well culture inserts (ibidi GmbH, Gräfelfing, Germany) was employed to initially determine cell migration rates. Seventy μL of cell suspension at a concentration of 2 × 10^5^ to 3 × 10^5^ melanoma cells per mL were seeded in each well, incubated at 37 °C under 5% CO_2_ condition overnight, and infected with *Salmonella* 100 M.O.I. for 90 min. Culture inserts were carefully removed and each well was washed with sterile PBS prior to the addition of fresh serum-free medium containing 50 μg/mL gentamicin. Photomicrographs were taken under 100× magnification at 10 and 21 h post treatment; migration distances were measured and expressed as percent wound closure relative to PBS control. The second approach for cell migration was a transwell assay using 8.0 μm Millicell^®^ Hanging Cell Culture Inserts (MilliporeSigma, Burlington, MA, USA). Cells were pretreated with either PBS or *Salmonella* 100 M.O.I. prior to seeding on the upper chamber of the culture insert placed on a 24-well plate. After 24 h incubation, the cells were fixed with 3% paraformaldehyde for 5 min, stained with 1 µg/mL 4′,6-diamidino-2-phenylindole (DAPI) for 2 min in the dark, and then the cells from the upper chamber that did not migrate were wiped using a cotton swab. Consequently, the cells were counted under a fluorescence microscope (Inverted, Olympus CKX41) at 200× magnification (Olympus LCAch N 20×/0.40 na objective at 3.2 mm working distance).

### 4.4. Constitutive Akt Expression

Melanoma cells (B16F10 and K1735) were prepared at 70–80% confluency in 6-well plates and transiently transfected with myr-Akt plasmid using lipofectamine 2000 (Invitrogen, Carlsbad, CA, USA) at a ratio of 1:2. The medium was changed after 16 h with serum-supplemented DMEM and re-incubated for another 24 h at 37 °C under 5% CO₂ condition prior to reseeding for subsequent experiments.

### 4.5. Macrophage Polarization

Melanoma cells (B16F10 and K1735) were pre-treated with *Salmonella* at 100 M.O.I. for 90 min prior to co-culture. After *Salmonella* treatment, the cells were washed with sterile PBS, and then the medium was replenished with 50 μg/mL gentamicin in appropriate serum-free medium (IMDM or DMEM for WEHI-3 or RAW 264.7 cells, respectively). WEHI-3 or RAW 264.7 cells were incorporated into the melanoma culture using 0.4 µm transwell hanging cell culture inserts, and the co-culture was incubated for 16 to 24 h thereafter. Subsequently, the macrophages and culture supernatant were separated for Western blotting and ELISA, respectively.

### 4.6. Enzyme-Linked Immunosorbent Assay (ELISA)

Filtered culture supernatants from PBS- or *Salmonella*-treated B16F10 and K1735 cells were collected after 24 h incubation. Supernatants were then analyzed for the presence of HMGB1 using a Mouse HMGB1 ELISA test kit (Bioassay Technology Laboratory, Shanghai, China) and TGF-*β*1 using a Mouse TGF-β1 ELISA kit (Arigo Biolaboratories, Hsinchu City, Taiwan) performed according to the manufacturer’s instructions. Furthermore, IL-1*β* from culture supernatants of WEHI-3 cells was determined using a Mouse IL-1*β* ELISA Set (BD Biosciences Pharmingen, San Diego, CA, USA) according to the manufacturer’s instructions with minor modification.

### 4.7. Animal Study

C57BL/6 and C3H/HeN mice were acclimated in the laboratory animal room for 1 week, provided clean water, and fed a normal diet ad libitum. The experimental protocol was carried out in accordance with all the relevant regulations on the ethical use of animals in research as approved by the Laboratory Animal Care and Use Committee of National Sun Yat-sen University, Kaohsiung 804, Taiwan (Permit No. 10801).

#### 4.7.1. Survival Analysis

C57BL/6 mice were injected with *Salmonella*-treated or PBS-treated B16F10 cells (2 × 10^5^ cells per mouse) via the lateral tail vein. In the same manner, C3H/HeN mice were injected with *Salmonella*-treated or PBS-treated K1735 cells (5 × 10⁵ cells per mouse). The mice were monitored starting day 1 post-tumor inoculation and the mortality rate was then recorded (Figure S4E).

#### 4.7.2. Assessment of Lung Metastasis and Macrophage Infiltrations

In a separate study, C57BL/6 and C3H/HeN mice were injected, as described in the previous clause, with *Salmonella*-treated or PBS-treated B16F10 and K1735 cells, respectively. The mice were sacrificed 15 days (C57BL/6) or 30 days (C3H/HeN) post-tumor inoculation; the lungs were removed, weighed, and fixed in 4% paraformaldehyde. Fixed lungs were embedded in paraffin and then processed for tissue sectioning. Tissue sections were subjected to H&E staining and were histopathologically examined for the presence of tumor nodules or metastases at 100× magnification. Lastly, lung tissue sections were subjected to immunohistochemical staining using Anti-mouse SNAI1, Anti-mouse EMR1 F4/80, and Anti-mouse iNOS. Each IHC marker was examined at 200× and 400× magnification. In another separate experiment, 4 groups for each mouse model were prepared, namely, a PBS group, an SC group, a PBS+Anti-HMGB1 group, and an SC+Anti-HMGB1 group. Each mouse was injected in the same manner as described above; however, the last two groups received either PBS- or *Salmonella*-treated melanoma cells that had been supplemented with anti-HMGB1 neutralizing antibody prior to tail-vein injection. Likewise, the mice were sacrificed 15 (C57BL/6) or 30 days (C3H/HeN) post-tumor inoculation; the lungs were removed and weighed, and the visible tumor growths were carefully dissected and lysed with lysis buffer for Western blotting (Figure S4E).

### 4.8. Statistical Analysis

Quantitative data are presented as mean ± standard deviation (SD) and the difference between the mean from the two groups was determined using Student’s *t*-test. Statistical analysis for experiments involving more than two groups was done using analysis of variance (ANOVA) and the difference among the mean was determined using Tukey’s multiple comparison test. Survival analysis was assessed using the Kaplan–Meier survival curve and log-rank (Mantel–Cox) test. A *p*-value of less than 0.05 was considered statistically significant. All statistical analyses were performed using GraphPad Prism version 6 (GraphPad Software, La Jolla, CA, USA). All graphs were generated using the same software.

## 5. Conclusions

Our data suggest that *Salmonella* directly inhibited melanoma metastasis in both in vitro and in vivo models by suppressing the Akt/mTOR/SNAI1 signaling cascade. Moreover, *Salmonella* treatment increased HMGB1 secretion in melanoma, and the increased extracellular HMGB1 facilitated macrophage polarization into the M1-like phenotype, an inflammation-related mechanism tapped by *Salmonella* to further prevent metastasis. Taken together, our findings provide a mechanistic basis toward understanding the action of *Salmonella* in cancer immunotherapy.

## Data Availability

Data are available upon reasonable request.

## Supplementary Materials

The following are available online at https://www.mdpi.com/article/10.3390/cancers13122894/s1, Table S1: Antibodies used for Western blotting; Figure S1: The expression levels of SNAI1 and Akt/mTOR phosphorylation following *Salmonella* treatment in human A2058 melanoma cells; Figure S2: *Salmonella* induces HMGB1 secretion in melanoma; Figure S3: *Salmonella*-induced secretion of HMGB1 in melanoma upregulates iNOS expression in macrophages; Figure S4: Effects of *Salmonella* on metastasis and the M1-like macrophage marker in vivo; Figure S5: *Salmonella* did not affect TGF-*β*1 secretion in melanoma 24 h after treatment.
