# Supplementary material for: Salmonella Impacts Tumor-Induced Macrophage Polarization, and Inhibits SNAI1-Mediated Metastasis in Melanoma
Source: Cancers (Basel). 2021 Jun 9;13(12):2894. doi: 10.3390/cancers13122894 (PMC8230152; doi:10.3390/cancers13122894)

Supplementary Materials

Salmonella Impacts Tumor-Induced Macrophage Polarization, and Inhibits SNAI1-Mediated Metastasis in Melanoma

Christian R. Pangilinan, Li-Hsien Wu, and Che-Hsin Lee

Table S1. Antibodies Used for Western Blotting.

Antibody	TargetSize (kDa)	Host	Dilutions	Catalog No.	Company
β-Actin	43	Mouse	1:5000	A5441	Sigma-Aldrich, St. Louis, MO, USA
Phospho-Akt	~63	Mouse	1:500	sc-81433	Santa Cruz Biotechnology, CA, USA
Akt	~63	Rabbit	1:1000	sc-8312	Santa Cruz Biotechnology, CA, USA
Phospho-mTOR	289	Rabbit	1:1000	2983	Cell Signaling Technology, MA, USA
mTOR	289	Rabbit	1:1000	2974	Cell Signaling Technology, MA, USA
SNAI1	29	Rabbit	1:1000	sc-28199	Santa Cruz Biotechnology, CA, USA
HMGB1	25	Rabbit	1:1000	GTX101277	GeneTex, CA, USA
iNOS	130	Rabbit	1:2000	PA3-030A	Invitrogen, Carlsbad, CA, USA

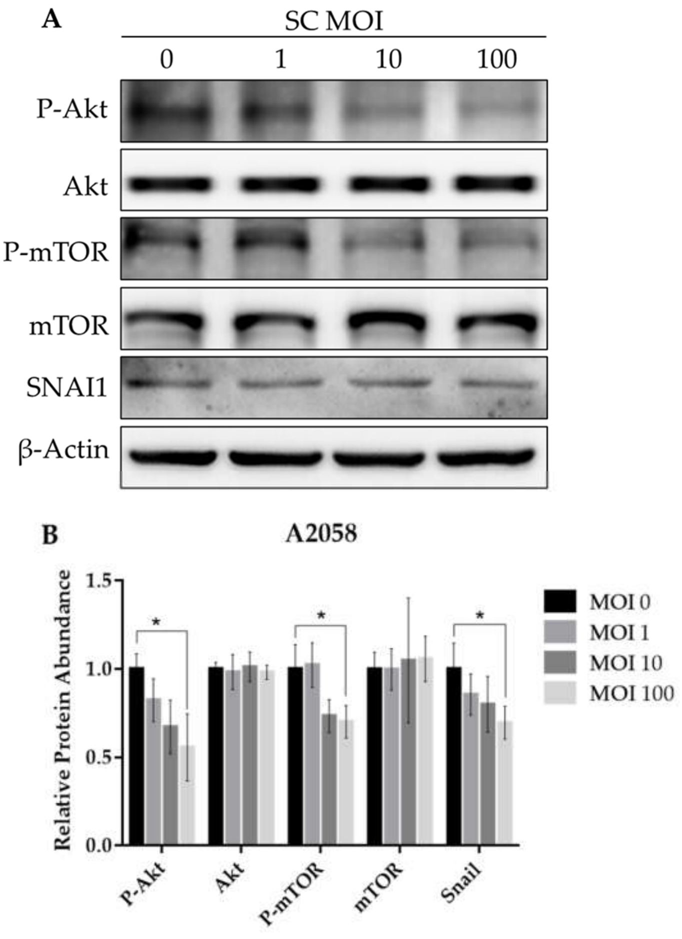

Figure S1. The Expression Levels of SNAI1 and Akt/mTOR Phosphorylation Following *Salmonella* Treatment in Human A2058 Melanoma Cell. **(A)** Western blot images showing SNAI1, Akt, P-Akt,

mTOR, and P-mTOR as affected by *Salmonella* treatment. **(B)** Quantified band intensities normalized to β-Actin, relative to 0 M.O.I. Human A2058 melanoma cells were seeded in 6-well plates with a seeding density of $5x10^5$. Cells were treated with varying doses of *Salmonella* from 0-100 multiplicity of infection (M.O.I.) for about 90 min.; cells were washed and cultured in serum-free DMEM with 50μg/mL gentamicin. After 24h, cells were lysed, and the lysates were subjected to western blotting. Data are presented as mean ± SD from three independent experiments. Statistical significance was calculated using Student's *t*-test; $*p < 0.05$.

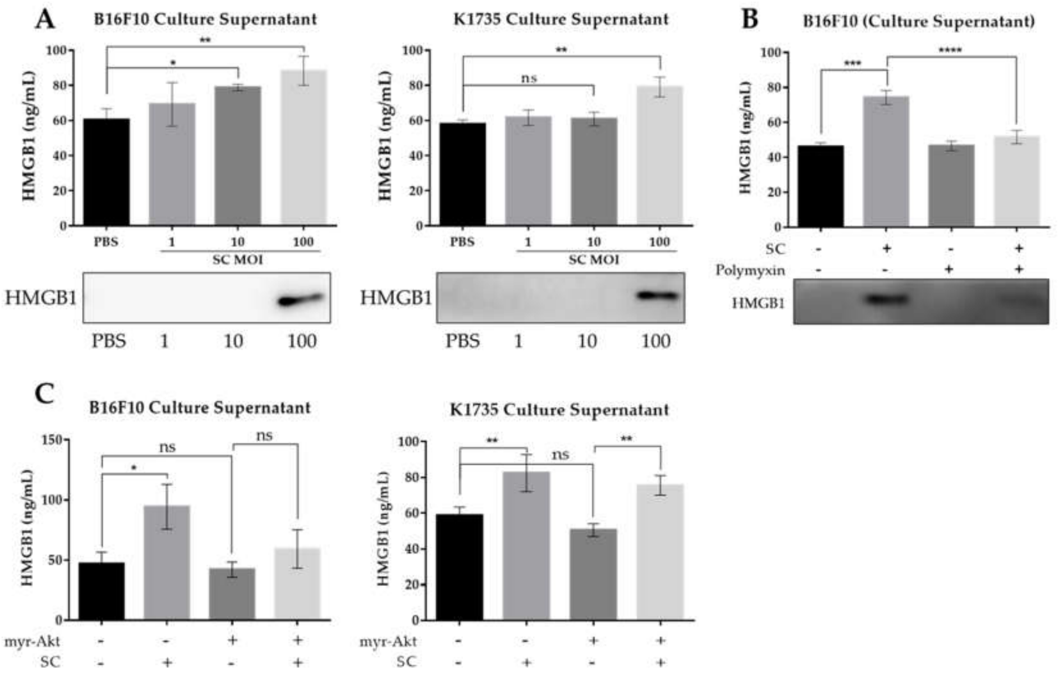

Figure S2. *Salmonella* induces HMGB1 secretion in melanoma. **(A)** Extracellular HMGB1 levels secreted by melanoma cells after *Salmonella* treatment; graphs show the data obtained from ELISA, while the figures below the graphs are from western blot. **(B)** Extracellular HMGB1 levels secreted by *Salmonella*-treated melanoma cells after LPS neutralization. **(C)** Akt Transfection Did Not Affect HMGB1 Secretion. Melanoma cells were seeded in 6-well plates, treated with 0-100 M.O.I. of *Salmonella*, and incubated for 24h; conditioned media was then analyzed for HMGB1 levels by ELISA and western blotting. Data are presented as mean ± SD from three independent experiments. . Statistical significance was calculated using Student's *t*-test (A), and ANOVA and Tukey's multiple comparison test (B-C); $*p < 0.05$, $**p < 0.01$, $***p < 0.001$, $****p < 0.0001$.

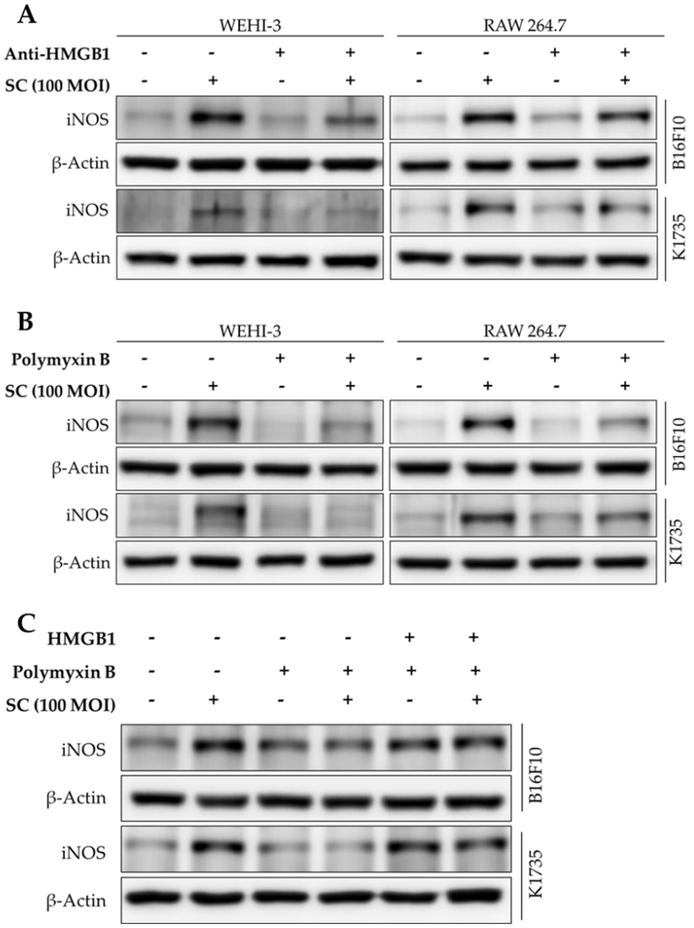

Figure S3. *Salmonella*-Induced Secretion of HMGB1 in Melanoma Upregulates iNOS expression in Macrophages. **(A)** Western blot images showing iNOS expression in WEHI-3 and RAW 264.7 cells as affected by Anti-HMGB1 neutralizing antibody (2.5 μg/mL). **(B)** Western blot images showing iNOS expression in WEHI-3 and RAW 264.7 cells as affected by Polymyxin B (10 μg/mL). **(C)** Western blot images showing rescued iNOS expression in WEHI-3 and RAW 264.7 cells following recombinant HMGB1 treatment in LPS-neutralized co-culture system.

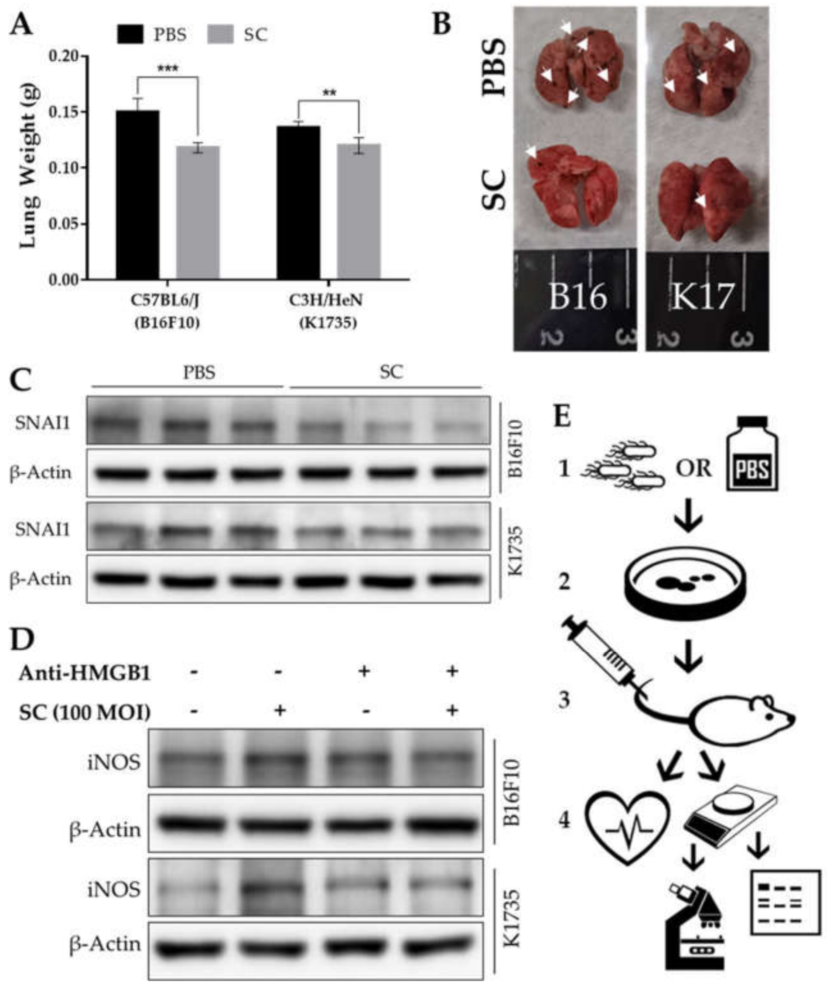

Figure S4. Effects of *Salmonella* on Metastasis and M1-like Macrophage Marker in Vivo. **(A)** Lung weight after 15- and 30-days post tumor injection with B16F10 and K1735 cells, respectively; n=5. Lung weight data was presented as mean ± SD. ** $p < 0.01$, *** $p < 0.001$. **(B)** Visible metastatic tumor growth. **(C)** Western blot images showing SNAI1 protein levels from lung metastatic lysates. **(D)** Western blot images showing iNOS protein levels from lung metastatic lysates. **(E)** Schematic representation of the tail vein assay for lung metastasis. (E.1) PBS or *Salmonella* (SC) preparation; SC was first cultivated in LB broth ≤16 hr prior to use. (E.2) The overnight culture of melanoma cells on 6-well plates, at 70-80% confluency, were treated with either PBS or 100 M.O.I. of *Salmonella* for about 90 min. Subsequently, the cells were washed 2x with PBS and the fresh DMEM free medium with 50 μg/mL gentamicin was added into each well, and then incubated for 24h at 37°C, 5% CO_2. (E.3) The cells were trypsinized, centrifuged and resuspended with fresh free medium; the number of cells per mL was measured and then diluted to reach the desired cell concentration prior to injection via the lateral tail vein. A separate mice cohort were prepared for survival, another for histopathology, and another for western blotting. (E.4) The mice for survival cohort were monitored daily to determine mortality. The mice for both histopathology and western blotting cohort were sacrificed at 15 (C57BL/6J) or 30 days (C3H/HeN).

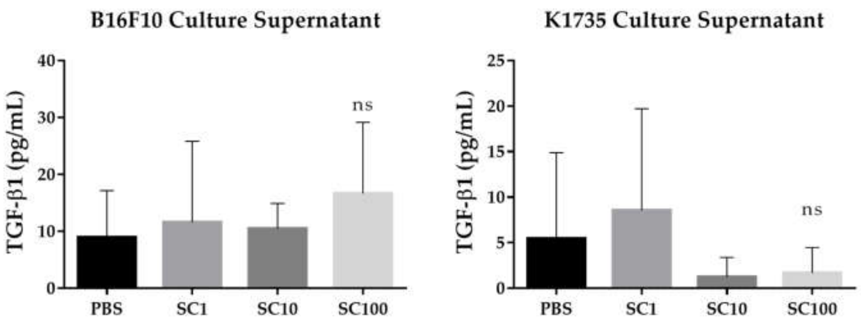

Figure S5. *Salmonella* did not affect TGF-β1 secretion in melanoma, 24 h after treatment. Melanoma cells were seeded in 6-well plates, treated with 0-100 M.O.I. of *Salmonella*, and incubated for 24h; conditioned media was then analyzed for TGF- β1 levels by ELISA. Statistical significance was calculated using Student's t-test; ns, not significant.

Uncropped western blot images for main Figure 1

(A–C)

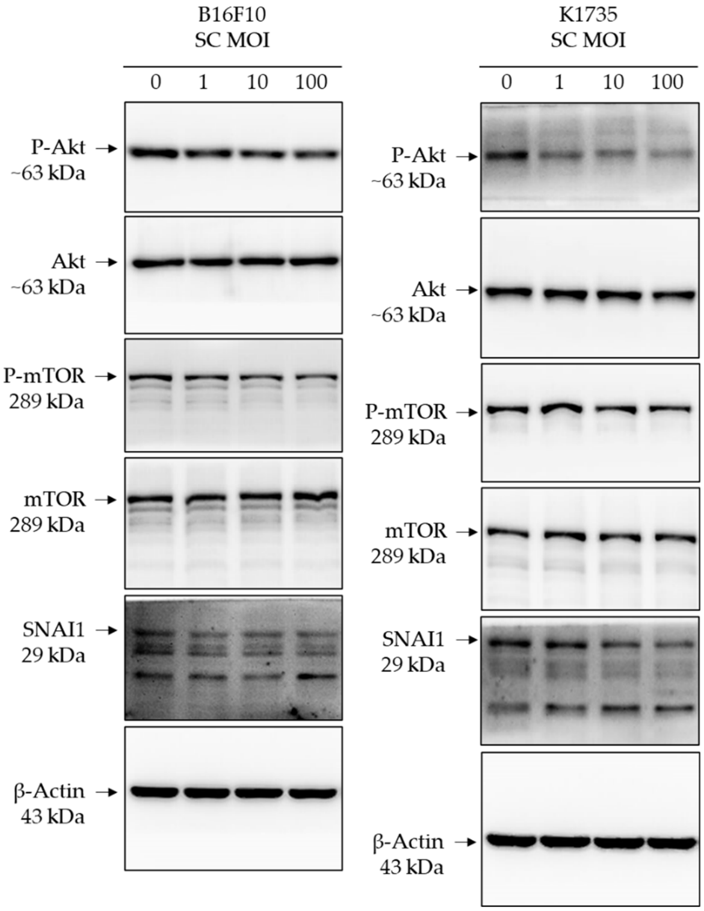

Uncropped western blot images for main Figure 3 (A)

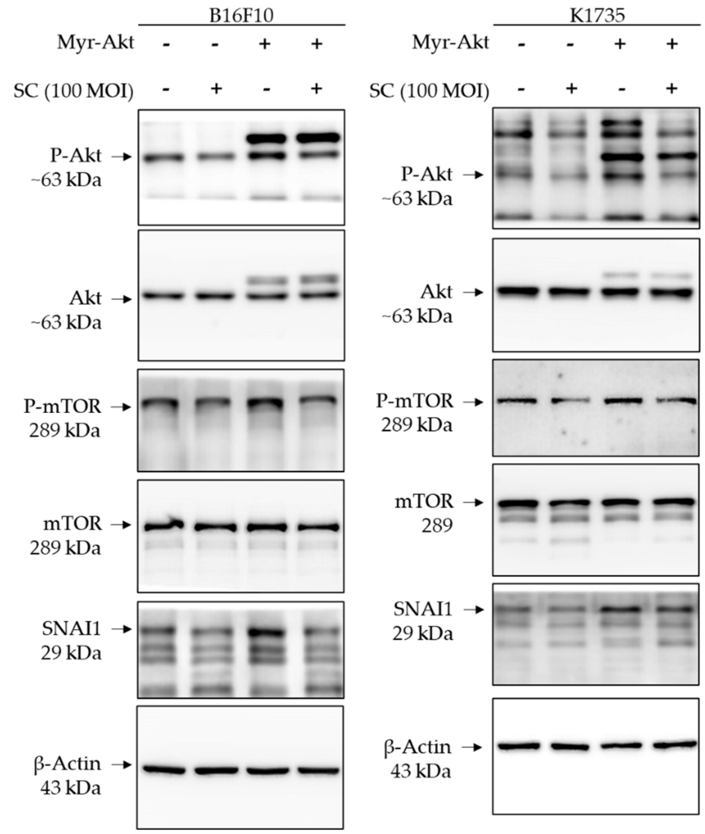

Uncropped western blot images for main Figure 4 (A)

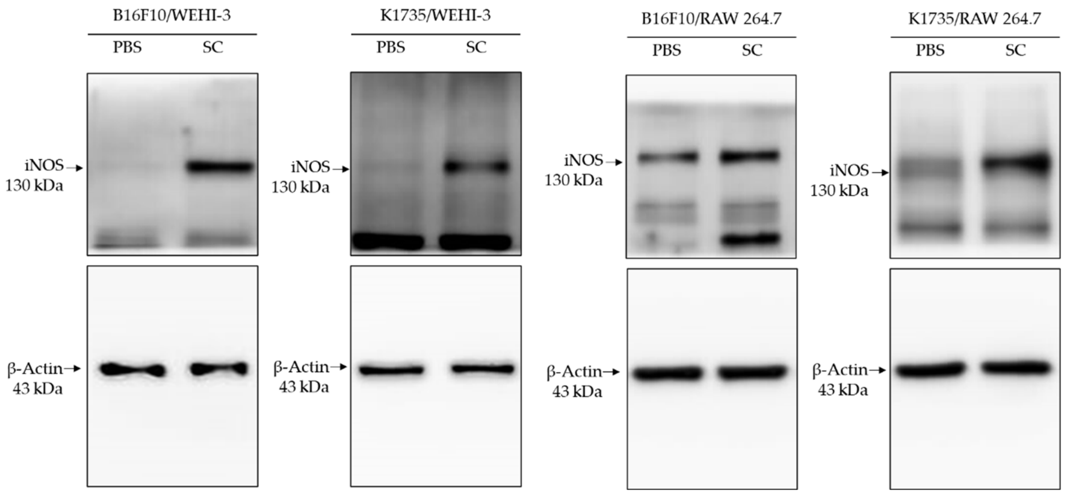

Uncropped western blot images for Figure S1

(A)

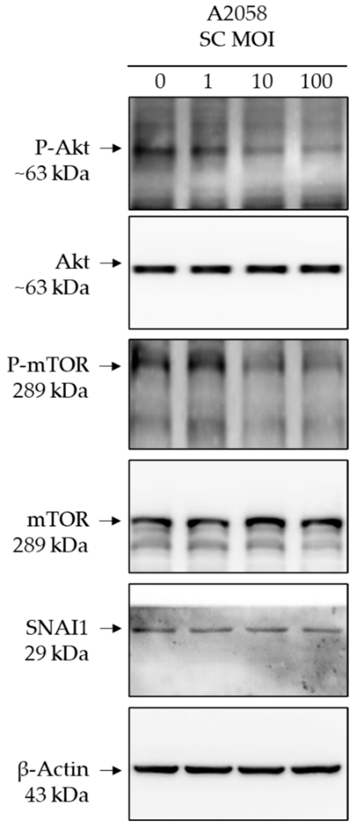

Uncropped western blot images for Figure S2

(A)

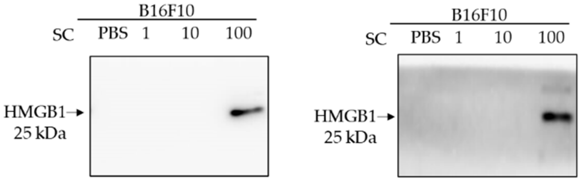

(B)

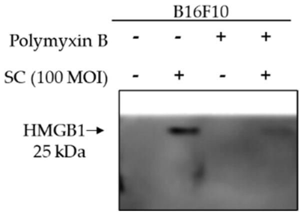

Uncropped western blot images for Figure S3

(A)

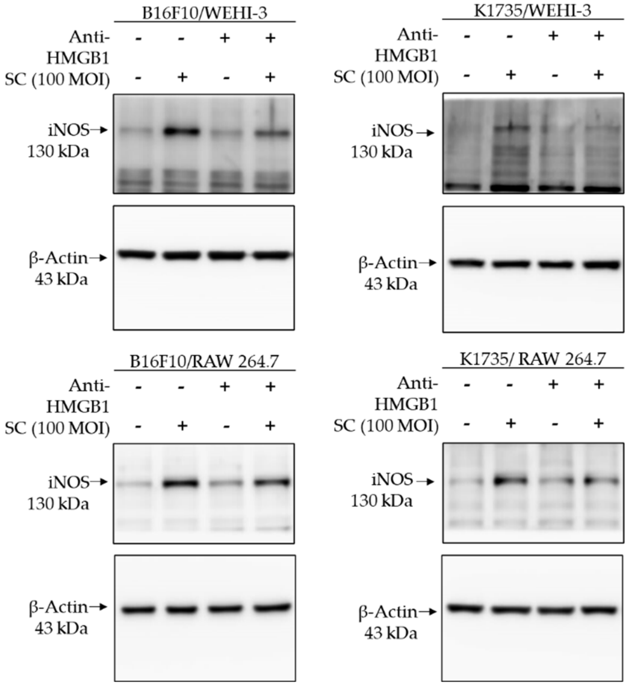

(B)

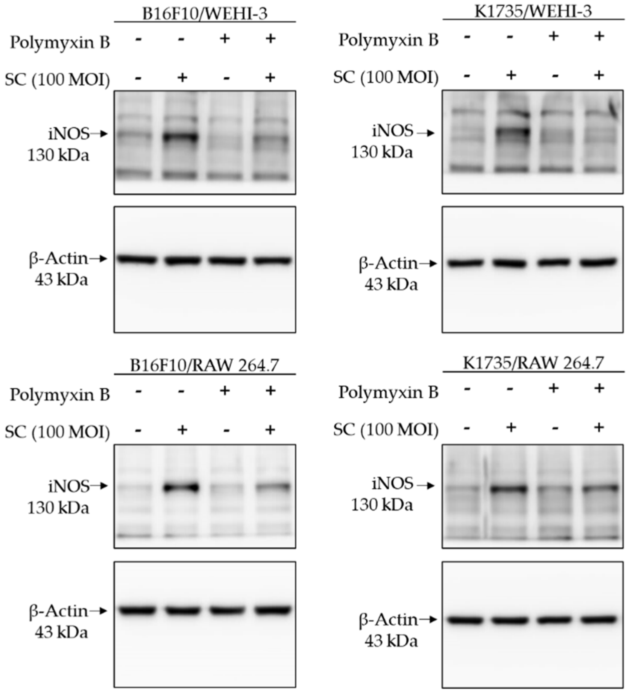

Uncropped western blot images for Figure S3 (continuation)

(C)

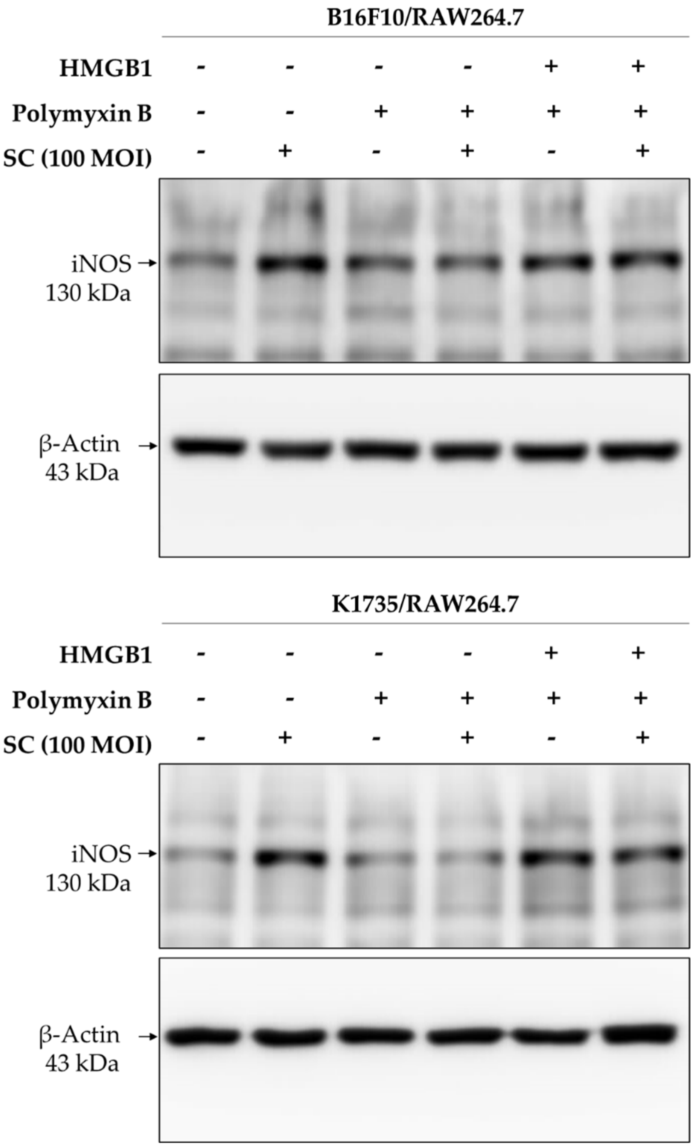

Uncropped western blot images for main Figure S4

(C)

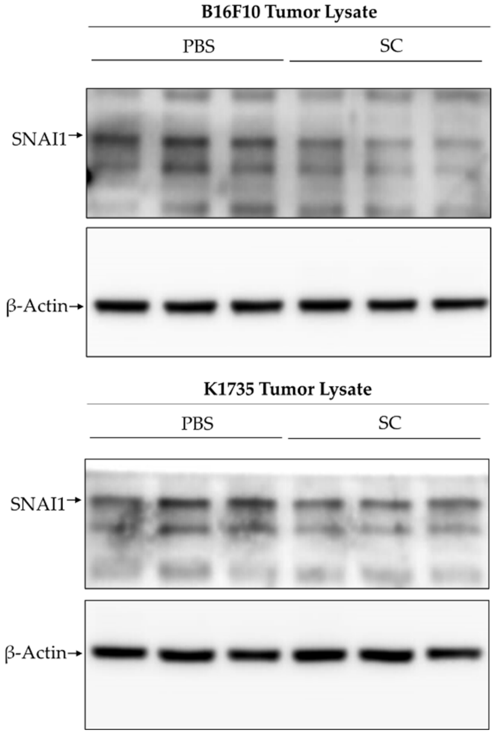

(D)

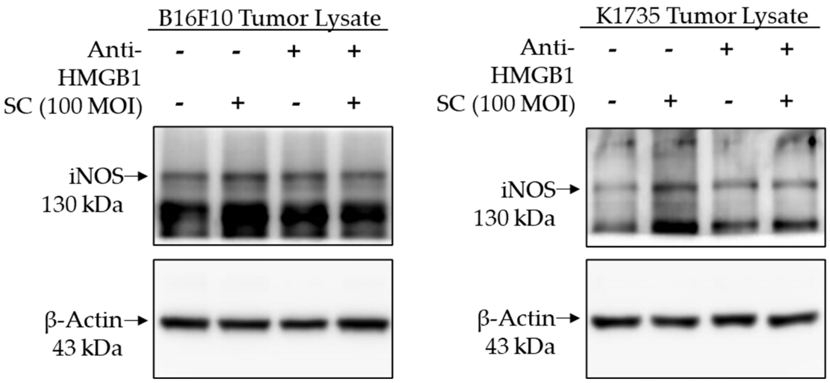

Supplement: Supplementary file 1 [file cancers-13-02894-s001.zip › cancers-1196732-supplementary.pdf]
